# Aggressive Pelvic Angiomyxoma in a Patient with Twin Pregnancy: Diagnosis, Treatment, and Clinical Complications in Light of the Literature

**DOI:** 10.3390/medicina59081417

**Published:** 2023-08-03

**Authors:** Carmen Imma Aquino, Raffaele Tinelli, Alessandro Libretti, Riccardo Bertinato, Renzo Luciano Boldorini, Michele Giana, Felice Sorrentino, Luigi Nappi, Valentino Remorgida, Daniela Surico

**Affiliations:** 1 Department of Gynecology and Obstetrics, “Maggiore della Carità” Hospital, University of Piemonte Orientale, 28100 Novara, Italy; c.immaquino@gmail.com (C.I.A.); libretti.a@gmail.com (A.L.); riccardo.bertinato.1991@gmail.com (R.B.); michele.giana@uniupo.it (M.G.); valentino.remorgida@uniupo.it (V.R.); daniela.surico@med.uniupo.it (D.S.); 2 Department of Translational Medicine, University of Piemonte Orientale, 28100 Novara, Italy; 3 Department of Obstetrics and Gynecology, “Valle D’Itria” Hospital, Martina Franca, 74015 Taranto, Italy; 4 Department of Pathology, “Maggiore della Carità” Hospital, University of Piemonte Orientale, 28100 Novara, Italy; renzo.boldorini@med.uniupo.it; 5 Department of Medical and Surgical Sciences, Institute of Obstetrics and Gynecology, University of Foggia, 71121 Foggia, Italy; felice.sorrentino.1983@gmail.com (F.S.); luigi.nappi@unifg.it (L.N.)

**Keywords:** angiomyxoma, pregnancy, cancer, soft mass

## Abstract

(1) Background: Aggressive angiomyxoma is a mesenchymal cancer that is rare during pregnancy. It is a neoplasm that relapses and infiltrates the nearest structures. Our aim is to evaluate the management and outcomes of an observed case, in light of the current literature. (2) Methods: We observed this condition at the “Maggiore della Carità” Hospital in Novara (Italy) in a patient with an initial twin pregnancy and a suspected pelvic mass. The words “angiomyxoma” and “pregnancy” were searched on the main online scientific search sources (PubMed, Google Scholar, Scopus, WES, and Embase, etc.). (3) Results: The patient underwent surgery with a complicated follow-up, but recent negative controls. We analyzed the literature about the topic and found only 24 similar clinical cases. (4) Conclusions: Considering the current literature, it is useful to assess an aggressive angiomyxoma in the differential diagnosis of soft masses in pregnant women. The treatment of choice is surgical excision, and vaginal delivery is feasible. The therapeutic decision depends on each case.

## 1. Introduction

Angiomyxoma is a rare mesenchymal tumor characterized by a possible infiltration of the nearest tissues: it is more frequent in women, and in the third and fourth decade of life [1,2]. The most interested anatomical sites are: vulva, vagina, pelvis, and perineum. It is a neoplasm that is prone to misdiagnosis and relapse (30% of cases) [3]. In general, its clinical presentation is asymptomatic and, initially, the neoformation is painless at the level of the vulva or vagina, with a variable size observed at the beginning of gestation, which then increases during the following months. The main differential diagnoss are: Bartholin gland cyst, lipoma, vaginal wall cyst, spindle cell lipoma, myxoid neurofibroma, intrauterine myxoma, and myxoid liposarcoma [2,4,5,6]. 

Our aim is to describe the clinical management of this rare cancer in a patient with a twin pregnancy who underwent surgical excision during gestation, considering the current literature.

## 2. Materials and Methods

We observed this condition at the “Maggiore della Carità” Hospital in Novara (Italy) in a pregnant patient with a suspected pelvic mass. 

The words “angiomyxoma” and “pregnancy” on the main online scientific search sources (PubMed, Google Scholar, Scopus, WES, and Embase, etc.) highlighted 128 results. Including case reports, case series, cohort studies, and meta-analyses in English, we reviewed 24 cases about this topic. We evaluated the published research, from the first study resulted in 1995 until today, with our described clinical case (Table 1). 

The study conformed to the Ethical Guidelines of the Helsinki Declaration. The patient gave informed consent for the use of her clinical data for research purposes. The review follows PRISMA Checklist 2020 indications.

## 3. Results

### Our Clinical Experience: The Case Report

Firstly, we described our clinical experience about this condition, and after, we reviewed the current literature, including our case (Table 1). 

A 30-year-old patient with a silent history, except subclinical hypothyroidism, came to the attention of the gynecologist for her first obstetric control. The pregnancy was a spontaneous dichorionic diamniotic gestation, without significant clinical events.

**Figure 1:**
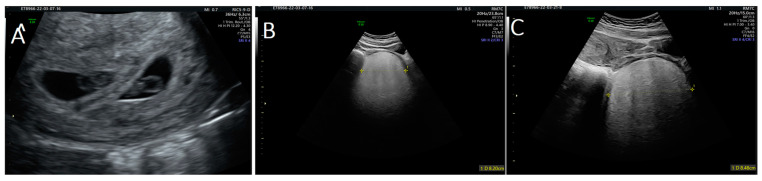
Ultrasound images of: (**A**) twin pregnancy; and (**B**,**C**) angiomyxoma.

During the first visit at 10 weeks of gestation, a transvaginal ultrasound revealed a hypervascularized and inhomogeneous pelvic formation of about 10 cm in maximum diameter (Figure 1), which was not present at the last gynecological visit performed the previous year. Our patient had no symptoms until this ultrasound check.

**Figure 2:**
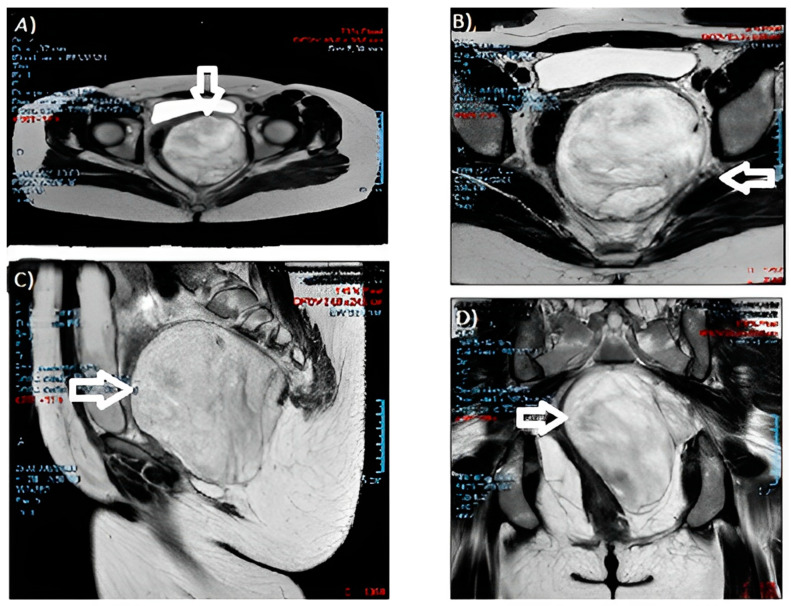
MRI images of the angiomyxoma: (**A**,**B**) cross-sections; (**C**) sagittal section; and (**D**) coronal section. (The arrow indicates the neoformation).

During the gynecological examination, a swelling of the left posterolateral vaginal wall with an antero-deviated portio was noted. The gynecologist performed further checks. Blood markers resulted negative. A protruding pelvic mass was described from the left perineal plane to the ischiorectal fossa on abdominal magnetic resonance imaging (MRI) (Figure 2), with dimensions of 10 × 10 × 11 cm and cottony content, hyperintense in T2, without a restriction of diffusivity. The neoformation compressed and contralaterally displaced the levator ani muscle and the wall of the rectum, anteriorly the bladder and the vaginal canal, with a cranial dislocation of the uterus. There were no appreciable signs of the infiltration of contiguous structures and no fluid flaps or lymphadenopathies. The growth and description were suggestive of an angiomyxoma. 

**Figure 3:**
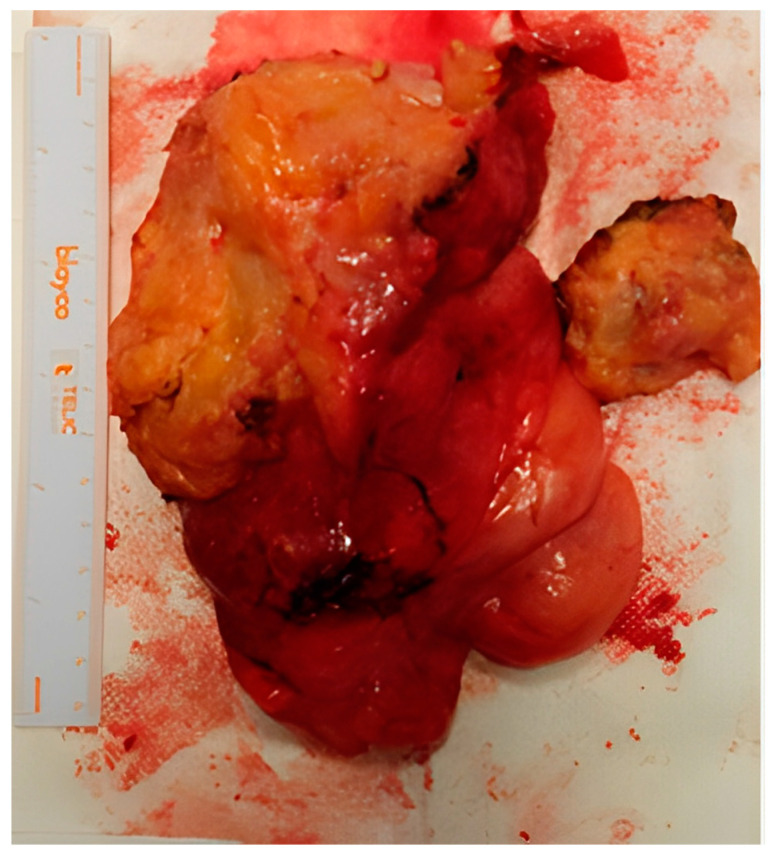
Macroscopic appearance of the aggressive angiomyxoma after surgery.

The clinical case was examined in a multidisciplinary setting with indication for surgery, due to the symptomatic increase and the patient’s severe sexual discomfort. Considering the growth of the neoformation and the patient’s will to be surgically treated, despite the possible risks and complications, the excision was vaginally performed at 13 weeks and 6 days of gestation (Figure 3). Under spinal anesthesia, an incision was made in the middle–lower third of the left vaginal wall for about 4 cm, in correspondence with the known swelling. The capsule appeared to be lardaceous and yellowish-white in color. At the end of the procedure, the formation was completely removed and sent for an extemporaneous histological examination: “myxo-lipomatous mesenchymal neoplasia without evident mitoses”. A rectal test with methylene blue was negative, the blood loss was <50 mL, and the fetal heartbeats were regular for both fetuses. 

After the definitive histological examination, the description was of an aggressive angiomyxoma, with fibroadipose tissue weighing 362 g and measuring 15 × 11 × 4.5 cm, yellowish with whitish streaks composed of adipose tissue, a myxoid appearance, and seeded by bands of loose collagen, including a modest cellularity, partly fused and partly stellate elements with a focal perivascular distribution, and a low mitotic index (<1/10 HPF). The immunophenotype was: actin +, desmin +, estrogen +/−, progesterone +/−, CD34+, S100−, MDM2−.

The patient was discharged home on the fourth postoperative day, with a regular course. The pregnancy was ongoing without complications. The recommendation was to perform a pelvic MRI and abdomen/chest computer-assisted tomography (CT) with contrast agent after delivery.

On the 10th postoperative day (at 14 weeks and 1 day of gestation), the patient came to the emergency room due to the appearance of hyperpyrexia and abdominal pain. Upon investigation, blood tests showed raised inflammation indexes. A vaginal ultrasound described a left pararectal dysomogeneous area of 8 × 5.7 × 4.1 cm. In a speculum examination, no abnormal genital discharge or signs of dehiscence were noted. There were no obstetric complications. She was therefore hospitalized with the diagnosis of a pelvic hematoma, and subjected to intravenous antibiotic therapy with piperacillin/tazobactam (4.5 g for three times/day). During her hospitalization, she was subjected to urine tests, urine cultures, and vaginal swabs with negative results. The progressive reduction in inflammation indexes, the resolution of her pelvic-abdominal pain, and the regression in temperature were observed. Daily fetal monitoring was regular. At the discharge visit, there was a dimensional reduction in the left pararectal suspected area (4 × 2.5 cm), with the presence of an inflammatory vaginal polyp of 1.5 × 0.5 cm between the middle and the distal third of the vaginal wound. She was discharged home on the 6th day of hospitalization, on the 16th post-operative day, with oral antibiotic therapy (amoxicillin/clavulanic acid 1 g for three times/day for another six days) and a scheduled follow-up visit for two months after the operation. Therefore, an elective cesarean section was planned at 38 gestational weeks of gestation, with a contextual excision of the suspected inflammatory vaginal polyp. The second and third trimester ultrasounds resulted in regular biometrics and anatomy for both fetuses.

At 36 weeks and 4 days of gestation, she arrived in the obstetric emergency department for the discharge of amniotic fluid. Upon examination, the PROM (premature rupture of membrane) test resulted positive, and at the obstetrical visit, the cervix was posterior, closed, with 20 percent effacement, and resistant. The twins were both cephalic and had regular amniotic fluid (the amniotic fluid deepest pockets were 4 and 5 cm, respectively, for the two fetuses). The CTG was normally reactive for both fetuses, with no perceived contractile activity. The vagino-rectal swabs were negative.

The patient tested positive at the routine SarsCOVID2 swab. She was therefore hospitalized for pPROM (preterm premature rupture of membrane) and subjected to short-cycle corticosteroid prophylaxis and antibiotic therapy, as per a protocol with Cefazolin 2 g and Azithromycin 500 mg. After two days, she was then subjected to a cesarean section and the contextual excision of the vaginal polyp, with a final blood loss of 400 mL. At the delivery, we reported the birth of a male newborn weighing 2740 g with Apgar score 8–9, and a male newborn weighing 2620 g with Apgar score 8–9.

The patient was discharged on the fifth post-operative day, with a regular course and two healthy children. Follow-up of the previous diagnosis of aggressive angiomyxoma demonstrated a normal CT and MRI.

## 4. Discussion

As emerged from our experience, and confirmed in the literature, the aspect of an aggressive angiomyxoma is often like a benign neoformation, such as lipomas or cysts, with a sort of soft, compact texture [2,4,5,6] and lobulated or spherical form, with an undefined capsule. The aggressivity of this type of tumor is usually limited to the nearest tissues, but some rare cases of pulmonary and lymph nodal metastasis have been reported [24,25,26,27].

Our patient had no symptoms until her first obstetric ultrasound check: despite the precious information of this technique, the gold standard remains a pelvic RMI and CT scan as an extension study. The images were similar to multiple twisted strip shadows, with “whirlpool” or “stratified” changes [28].

At the pathological level, the section could appear translucent, gray, white, or brown, with a homogeneous jelly-like consistency, cystic, and bleeding areas. Hypocellular tissues with myxoid stroma, edematous mucoid, collagen matrix, and stellate or spindle cells plus an elongated cytoplasm and abnormal vascularization are pathognomonic. The immunohistochemical examination is based on positivity for vimentin, desmin, CD34, hormone receptors (estrogens and progesterone, up to 80% of cases), S100, and a low Ki67 (<1% tumor cells) [2].

During pregnancy, the literature reports an important growth action of hormones, which should not be underrated [2]. As in our case, the suggested treatment is a surgical excision of the neoformation, trying to obtain radicality with free margins. If a neoplasm occurs during pregnancy, it could be possible to decide to treat it after delivery if there are no symptoms or discomfort [2].

In our case, the goal of surgery was reached, but a pelvic hematoma occurred as a complication and an inflammatory polyp was observed at the surgical site. The patient was then treated, with the resolution of these conditions. Vaginal delivery was not contraindicated [2], but a cesarean section was successfully performed after preterm premature membrane rupture, which was preferred due to the risk of bleeding and vulnerability of the treated perineal area. 

In the literature, there are suggestions for treatment, with hormonal therapy as agonists of gonadotropin-releasing hormone (GnRh) also as an adjuvant drug, but there are no clear data on its use [29,30]. 

Despite radicality, it is very important to adhere to a regular imaging follow-up to diagnose eventual recurrences. It was suggested to our patient to perform a pelvic MRI and an abdomen/chest CT with contrast agent after delivery and 6 months later.

## 5. Conclusions

The uniqueness of our work is not only for the analysis of the pathological management, but also for the presence of a complex, ongoing twin pregnancy and the observed sequelae of the treatment. To our knowledge, this is the most updated review on the topic [1,2,3,4,5,6,7,8,9,10,11,12,13,14,15,16,17,18,19,20,21,22,23,24,25,26,27,28,29,30]. The variety of the possible body locations, the association with pregnancy, and the few cases known in the literature make it difficult to formulate unique guidelines on this subject.

This article could be helpful for all health practitioners that have to manage pelvic neoformations and make complete diagnostic hypotheses.

In fact, aggressive angiomyxoma is a very rare disease, but it is necessary to include it in differential diagnoses in the case of pelvic neoformations, also during pregnancy. Surgical excision is its key therapy. Follow-ups through imaging (preferable MRI) are highly suggested to recognize recurrence and treat it promptly, with an attention comparable to other oncological conditions subjected to recurrence [31]. 

Usually, its obstetric outcomes are very encouraging: the delivery route could be differently decided for each gestation. 

In the future, imaging techniques and therapeutic strategies could be implemented and diversify the treatment choice.

## Figures and Tables

**Table 1:** Review of the literature.

	Reference	Age (Years)	Clinical Presentation	Dimension and/or Gestational Age at the First Individuation	Site of Tumor	Treatment
1	Current case	30	Hypervascularized and inhomogeneous pelvic formation at ultrasound check	10 cm in maximum diameter at 10 weeks of twin gestation	Left perineal area	Surgical excision at 13 weeks and 6 days; after, cesarean section at term
2	Espejo-Reina 2022 [7]	36	Genital prolapse and bilateral ovarian teratomas	At 26 gestational weeks 7 × 3 × 3.5 cm; in puerpuerium, the mass was 7 × 7 × 7.5 cm	Vesico-vaginal space	Elective cesarean section, tumor exeresis in puerpuerium
3	Xu, 2020 [2]	32	Cancer increased during two subsequent gestations and decreased one year after last delivery	From 3 cm to 7 cm during pregnancies, after delivery was 5.3 × 5.1 × 4.2 cm	Right labium majus	Surgical excisions during the puerpuerium of the two pregnancies
4	Malukani, 2018 [4]	24	Vaginal blood loss, difficulty in walking, and pain in the abdomen and lower back	11.4 cm × 11.3 cm × 9.9 cm at 17 weeks of gestation	Vaginal fornix	Surgical removal, curettage of the abortion
5	Obi-Njoku, 2017 [8]	23	Incidental finding on ultrasound scan	1.2 × 1.5 × 1.3 cm with no increased vascularity during pregnancy	On the posterior wall of the bladder, close to the left vesico-ureteric junction	Rigid cystoscopy and transurethral resection of the bladder tumor (TURBT) under epidural anesthesia
6	Orfanelli, 2016 [9]	29	Pudendal swelling with an increasing neoformation	2 cm at 20 weeks, 7 cm at 37 weeks	Right labium majus	Caesarean section with surgical resection (at 39 weeks)
7	Sampaio, 2016 [5]	25	Vaginal swelling, dyspareunia, and genital bleeding	11 cm at 9 weeks, 12 cm at 13 weeks	Vaginal fornix	Surgical resection (at 13 weeks) and full-term vaginal delivery
8	Zangmo, 2016 [10]	21	Recurring palpable mass, without pain	Dimension of an almond at 20 weeks, 8 cm at 32 weeks, then 15 cm at 37 weeks. Increased to 18 cm at 6 weeks postpartum	Right labium majus	Caesarean section (at 38 weeks) and surgical excision after two weeks
9	Ashraf, 2014 [11]	24	Pain with large pedunculated lobulated growth	Described for the first time at 16 weeks, was 30 cm at 20 weeks	Right labium majus	Surgical resection (at 20 weeks) and caesarean section (at 37 weeks)
10	Goyal, 2014 [12]	25	Painless, pedunculated, and soft mass	Diagnosed at 12 weeks, was 8 cm at 18 weeks	Left labium majus	Excision (at 18 weeks) and full-term delivery
11	Sinha, 2014 [13]	43	Difficulty in walking	Gradually increased for 10 years, suddenly during pregnancy and postpartum. At 9 months after the cesarean section, it was 55 cm	Left labium majus	Caesarean section and postpartum surgical resection
12	Dahiya, 2011 [14]	32	Swelling and discomfort (probably a recurrence)	At 16 weeks, 3 × 4 cm	Perineal region	Surgical excision
13	Aye, 2009 [15]	22	The patient had experienced the diagnosis and treatment of an angiomyxoma the year before, with no detectable effect After months, new vulvar discomfort	3.1 cm at 8 weeks, 5.1 cm at 32 weeks	Right vestibule	Pregestational surgery and GnRH analogue therapy. Full-term caesarean section (at 38 weeks). At 6 weeks after delivery, the mass was reduced in size to 3.6 × 3.1 × 2.9 cm with a multimodal approach
14	Mandal, 2008 [16]	22	Large pedunculated swelling mass with an ulcerated area	At 16 weeks, 7 cm of maximum diameter	Left labia majora	Surgical excision during pregnancy
15	Bagga, 2007 [17]	25	Soft, painless swelling	2 cm at 12 weeks and 4 cm at 16 weeks	Right labia majus	Surgical excision with massive bleeding (at 16 weeks), and full-term induced delivery
16	Lepistö, 2007 [18]	27	Unusually large belly	At 12 weeks, a large retroperitoneal jelly neoformation	Abdomen, in the lower pelvis, near to the uterus	Laparotomies for surgical excision at 16 weeks and in post-partum, with residual tumor. Spontaneous labor at term
17	Han-Geurts, 2006 [19]	31	Swelling during her first pregnancy 4 years before. This mass increased during the second pregnancy	Larger in the second pregnancy than in the first	Left gluteal area, paravaginal, and pararectal mass	Biopsy and Posterior exenteration+ neoadjuvant radiotherapy with 60 Gy (not any other details)
18	Han-Geurts, 2006 [19]	34	Swelling aspect	At 30 gestational weeks	Left and right labia majora	Vulvectomy (not any other details)
19	Han-Geurts, 2006 [19]	27	Mass	Early pregnancy	Right abdomen, in front of the bladder	Excision during caesarean section (not any other details)
20	Ribaldone, 2004 [20]	36	Mass	The neoformation grew extremely slowly before and during pregnancy, but very rapidly after the delivery (15 cm at last resection).	Right pelvic-perineal area	Surgical excisions, months after elective cesarean section
21	Wolf, 2003 [3]	32	Soft proliferation, suspected as massive condylomata acuminata	Diagnosed at 32 weeks; 3 × 4 cm at 36 weeks	Vulva, posterior commissura	Surgical excision (at 36 weeks) and vaginal at-term delivery
22	Smirniotis, 1997 [21]	38	Soft, bulky, palpable extraperitoneal swelling (suspected as regional haematoma)	Diagnosed during a follow-up after a recent cesarean section, dimension of 15 × 12 × 6 cm	Right lateral part of the pelvi	Exploratory laparotomy with resection, months after the cesarean section
23	Htwe, 1995 [22]	41	Soft, nontender mass	6 × 6 × 4 cm at 18 weeks	Left vulva	Surgical excision in the second trimester of pregnancy and full-term delivery
24	Fishman, 1995 [23]	37	Recurrence of mass	Pregestational treatment, 3 cm during pregnancy, to 40 cm in 3 years	Right vulva	Surgical excisions

## Data Availability

Not applicable.
